# Cutaneous Epithelioid Clear Cells Angiosarcoma in a Young Woman with Congenital Lymphedema

**DOI:** 10.1155/2013/931973

**Published:** 2013-09-03

**Authors:** Flore Tabareau-Delalande, Anne de Muret, Elodie Miquelestorena-Standley, Anne-Valérie Decouvelaere, Gonzague de Pinieux

**Affiliations:** ^1^Department of Pathology, Tours University Hospital, 37044 Tours, France; ^2^Department of Biopathology, Léon Bérard Center, 69008 Lyon, France

## Abstract

Angiosarcomas are rare aggressive neoplasms that can occur secondary to chronic lymphedema (Stewart-Treves syndrome). Although secondary angiosarcomas are commonly described after-mastectomy and/or after-radiotherapy, few cases have been reported in association with chronic lymphedema of congenital origin. We report the clinical, pathological, and cytogenetic findings in a case of cutaneous epithelioid clear cells angiosarcoma that occurred in a 21-year-old woman with hemibody congenital lymphedema. Surgical biopsies of the tumor mass revealed diffuse epithelioid proliferation of clear atypical cells, for which immunophenotyping highlighted the vascular differentiation. Despite *en bloc* resection of the tumor, the patient died of metastatic disease three months after diagnosis. This case illustrates the clinical and pathology characteristics of angiosarcoma that is a rare entity secondary to chronic lymphedema. It is the first reported case for which the *c-MYC* amplification status was assessed. The diagnostic value of this amplification should be further evaluated in this specific context.

## 1. Background

Angiosarcoma is an aggressive neoplasm that occurs in various soft tissues and visceral organs. Cases of angiosarcoma are rare and represent less than 1% of all sarcomas [1]. The most common sites (in decreasing order) are the skin, the breast, deep soft tissues, visceral organs, and bones. When the tumor occurs in the deep soft tissues, the preferential locations are the lower extremities, followed by the arms, the trunk, the head, and the neck [2]. The preferential locations for cutaneous angiosarcoma are the head and the neck, the limbs and the trunk [3]. Angiosarcoma can arise *de novo* (primary angiosarcoma) or secondary to either local irradiation or in patients with long-standing lymphedema (secondary angiosarcoma) [4]. Chronic lymphedema is usually acquired after radiotherapy and/or lymph node dissection for breast cancer. Less frequently, chronic lymphedema can be congenital, as in Milroy's disease. Secondary angiosarcoma occurring with chronic lymphedema forms part of the Stewart-Treves syndrome [5, 6]. This report provides clinical, pathology, and cytogenetics findings in an epithelioid cutaneous angiosarcoma occurring in a specific context of chronic lymphedema in a young woman with hemibody congenital lymphedema.

## 2. Case Presentation

A 21-year-old woman with right hemibody congenital lymphedema caused by hypoplasia of the lymphatic channels developed a huge tumor mass in her right groin. Lymphedema was diagnosed when she was 3 months old and progressively increased, affecting the right hemibody (arm, leg, and hemiface). She underwent many surgical interventions such as lymphatic aspiration, node autografts, lymphedema tissue excision, labiaplasty majora, and lymphovenous anastomosis to reduce the lymphedema and to treat complications. Despite compression, manual drainage, and pressure therapy, her lymphedema required several hospital admissions for intensive lymphatic drainage, with relative success. She was referred for a sensitive subcutaneous nodule in her right groin. Ultrasound showed a hypoechogenic heterogeneous tumor with liquid content, the size of which rapidly increased from 1.5 cm to 5.5 cm at the widest point in a few weeks. Tomodensitometry showed a heterogeneous, hypervascularized sub-cutaneous nodule. Skin punch biopsies were performed.

## 3. Pathology Findings

### 3.1. Punch Biopsy

**Figure 1:**
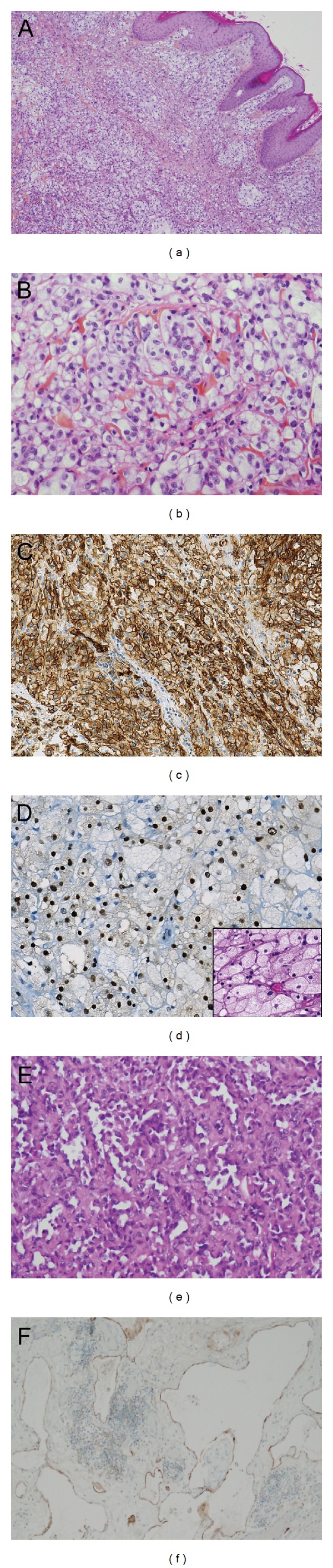


Low-power microscopy examination showed a poorly differentiated, dense proliferation of clear cohesive epithelioid cells, infiltrating the dermis and arranged in continuous compact sheets in a thin fibrous stroma (Figure 1(a)). High-power microscopy examination showed polygonal clear cells with an irregular nuclear outline and prominent nucleoli (Figure 1(b)). Mitotic activity was high. Immunohistochemistry study revealed that the epithelioid tumor cells only expressed CD31 (Figure 1(c)). Immunostainings for CD34, D2–40, factor VIII, cytokeratin AE1–AE3, CD45, HMB45, S100, and CD163 were negative. This immunophenotype in an epithelioid cell malignant tumor, in the context of chronic congenital lymphedema, suggested an epithelioid angiosarcoma. However, in the absence of clearly identified morphological vascular differentiation on this punch biopsy and in view of the therapeutic and prognostic implications of such a diagnosis, a surgical biopsy was also performed. The latter showed proliferation of the same poorly differentiated epithelioid clear cells in the dermis without epidermal infiltration. The tumor pattern was compact, composed of cohesive atypical ballooning round cells or with foamy cytoplasm and large vesicular nuclei. However, small clusters of erythrocytes were observed within the cytoplasm of epithelioid clear cells. At the deep level, tumor cells with a higher nucleocytoplasmic ratio and hyperchromatic nuclei lined some vascular spaces or channels (Figure 1(e)). In the hypodermis, tumor areas were composed of multilayered atypical endothelial cells forming papillary-like projections protruding within the vascular lumen. These morphological features confirmed the vascular origin of the proliferation. Significant areas of hemorrhage and necrosis were observed. Mitotic activity ranged from 20 to 25 mitoses per 10 high power fields. Both differentiated and poorly differentiated tumor cells expressed vascular markers (diffuse and strong intensity staining with CD31 and focal and moderate intensity staining with D2–40). Immunostainings for cytokeratin AE1-AE3, EMA, CD34, and PS100 were negative. A diagnosis of cutaneous high-grade epithelioid angiosarcoma (grade 3 of the FNCLCC classification) occurring with congenital lymphedema was made. The patient underwent extensive resection of the tumor with cutaneous abdominal flaps.

### 3.2. Surgical Specimen

**Figure 2:**
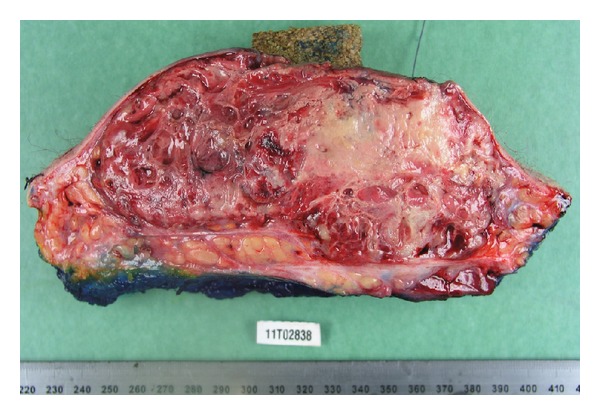


The tumor measured 13 × 9 × 5.5 cm and was of reddish lobulated appearance, with large areas of necrosis (Figure 2). It ulcerated the cutaneous flap and infiltrated the adipose tissues without muscle infiltration. Histologically, the tumor massively penetrated the dermis and hypodermis. As observed on the earlier biopsies, the proliferation was superficially poorly differentiated, composed of large solid epithelioid clear cells; some of them exhibiting xanthomatous-like features (Figure 1(d) inlet) but more deeply was better differentiated with hyperchromatic endothelial-like cells forming vascular slits hosting erythrocytes. Immunostainings for CD31, GLUT1, and ERG (Figure 1(d)) were strongly positive. Immunostainings for cytokeratin AE1–AE3, EMA, and CD34 were negative. The D2–40 immunostaining showed slight positivity in tumor cells and highlighted lymphatic ectasia in the hypodermis (Figure 1(f)).

### 3.3. Cytogenetics

We investigated *c-MYC* amplification, known to demonstrate a high level of amplification in secondary angiosarcoma (secondary to irradiation or chronic lymphedema) but not in primary angiosarcoma [7]. The fluorescence *in situ* hybridization (FISH) study performed on the biopsy material and on the surgical specimen (frozen samples) did not show any* c-MYC* amplification (data not shown). 

### 3.4. Outcome and Followup

Two months after surgery, the patient developed multiple lung metastases, revealed by significant pleural effusion. The patient died 3 months after diagnosis. 

## 4. Discussion

 We report a case of cutaneous congenital lymphedema-associated secondary angiosarcoma, the diagnosis of which was rendered difficult by its predominant poorly differentiated epithelioid clear cell morphology. Epithelioid appearance is more common in angiosarcomas arising in deep soft tissues [8]. In the skin, Bacchi et al. quite recently reported a series of 18 cases of epithelioid angiosarcomas. In this study, the authors documented a phenotypical tumor diversity in 7 cases. They thus described 5 different growth patterns, that is syncytial, nodular, solid, micronodular, and diffuse and three unusual microscopic subtypes, that is clear cells, plasmocytoid cells, and cells with “glassy” or granular cytoplasm [9]. The clear cells phenotype may notably simulate a carcinoma or a melanoma and lead to misdiagnosis. The xanthomatous-like features focally observed in the present case are very unusual. It may be also confusing on a fine-needle biopsy, especially since that histiocytes may express CD31. In the case we report here, the vascular differentiation was more obvious on the surgical specimens but remained essentially located in the very deep infiltrating areas of the mass. For this reason, a large sample may be recommended for histological typing and grading of these tumors. 

The positivity of a panel of vascular immunohistochemical markers, including CD31, CD34, and ERG, usually confirms the diagnosis. ERG is a highly sensitive marker of endothelial cells and vascular tumors. GLUT1 is mainly helpful in the differential diagnosis between malformative vascular lesions and capillary hemangioma, but its positivity has also recently been described in sarcomas and angiosarcomas exhibiting epithelioid features [10, 11]. 

 Secondary angiosarcoma may involve the skin, deep soft tissues, or deep organs. Cutaneous secondary angiosarcoma can develop in a context of chronic lymphedema (Stewart-Treves syndrome). More than 200 cases of cutaneous secondary angiosarcoma have been reported in the context of lymphedema of the upper extremity, occurring 10 to 20 years following radical mastectomy [3]. In contrast, secondary angiosarcoma occurring with congenital lymphedema is rare, and its pathogenesis is not clear. Only 19 cases of secondary angiosarcoma were found in the literature, each occurring in congenital lymphedema. Since the first case reported by Kettle in 1918, Offori et al. provided a review of 15 cases [12], and four additional cases have been described in the last fifteen years. All these cases were cutaneous secondary angiosarcoma. The clinical features of all these cases, including the present case, are summarized in Table 1. The average age of the patients was 33 years (range 2 to 85 years), 70% were females, and the interval to diagnosis was usually long, ranging from two months to three years (average 9 months, 9 cases not stated). Wide surgical excision was the primary therapy reported for all patients. One patient received adjuvant chemotherapy, and 4 patients had adjuvant radiation therapy. Histological findings were available for only 8 cases: two cases showed an epithelioid pattern (one pediatric case and the present case). Beside its secondary nature, the present case of angiosarcoma cumulated several criteria of poor prognosis, as described by Deyrup et al. [13]. These criteria were studied in a series of 69 cases of sporadic cutaneous primary angiosarcoma and showed that increased mortality was linked to age, anatomic locations (prognosis for trunk and extremities was worse than for head and neck locations), presence of tumor necrosis, tumor size over 5 cm, and epithelioid cytological features [13]. Moreover, tumor depth was correlated with the risk of local recurrence. In another study involving pediatric cases of cutaneous angiosarcoma, Deyrup et al. reported a disproportionate number of purely epithelioid form cells (9 out of 10 cases, including one case of cutaneous secondary angiosarcoma occurring with congenital lymphedema), compared to the cutaneous angiosarcoma occurring in adults (around 30% of epithelioid primary angiosarcoma) [14]. Surprisingly, the mortality was not increased for the children with cutaneous epithelioid angiosarcoma (mortality rate 40% in children versus 43% in adults) [14]. Half of the patients (5 of 10) developed metastases (bone, pleura, lung, liver, or soft tissue), of whom 3 had high-grade angiosarcoma, and 4 had epithelioid features. Four patients were alive without evidence of disease (with a followup of 20 months to 28 years).

 The molecular pathogenesis and cytogenetics of cutaneous secondary angiosarcoma in the context of chronic lymphedema are poorly characterized. Several authors have hypothesized that chronic lymphedema contributed to local immune disorders and to the development and progression of some angiosarcomas [15]. Manner et al. showed recurrent chromosomal changes in secondary angiosarcoma, which are not encountered in primary angiosarcoma [7]. In this study, of 22 cases (8 primary angiosarcoma; 14 secondary angiosarcoma) analyzed by comparative genomic hybridization array (CGH-array), 10 cases of secondary angiosarcoma (9 irradiated and 1 case associated with chronic noncongenital lymphedema) showed recurrent changes, whereas no recurrent changes were observed in primary angiosarcoma. The most frequent changes in secondary angiosarcoma were high-level amplifications on chromosome 8q24.21 (8 cases) followed by amplifications on chromosome 10p12.33 (6 cases) and amplifications on chromosome 5q35.3 (2 cases). The authors applied FISH in the chromosomal region 8q24.21 containing the *c-MYC* proto-oncogene. High-level gene amplifications of *c-MYC* were found in 55% (18/33 cases) of secondary angiosarcoma, including secondary angiosarcoma associated with chronic noncongenital lymphedema (number of cases not specified) and not in the patients with primary angiosarcoma (0/28 cases). The authors concluded that, although these tumors were not morphologically distinguishable, primary and secondary angiosarcomas were genetically different entities. In a recent series of 25 cases, Mentzel et al. confirmed the presence of an amplification of *c-MYC* in all cases of secondary angiosarcoma studied by FISH and showed that such an abnormality was lacking in patients with atypical vascular proliferation (16 cases studied) [16]. Immunostaining for *c-MYC* has been developed and might help to distinguish angiosarcoma from atypical vascular proliferation in difficult cases [17].

 The presence of *c-MYC* amplification may therefore be an additional diagnostic tool in the diagnosis of secondary angiosarcoma. Such amplification was absent in the case reported here. However, as this case of congenital chronic lymphedema was, to the best of our knowledge, the first in which a *c-MYC* amplification was sought, no conclusion can be drawn. The status of the *c-MYC* gene should therefore be further evaluated in the specific situation of chronic congenital lymphedema.

## Figures and Tables

**Table 1:** Clinical features of patients with cutaneous angiosarcoma associated with congenital lymphedema described in the literature (modified from Offori et al., 1993 [12] and Bernardi et al., 2009 [18] ).

Author	Sex	Age at diagnosis	Tumor site	Clinical appearance	Interval in diagnosis	Treatment	Survival
Kettle [19]	F	44 y	RL	Blue-red dislocation	Not stated	Amputation	Not stated
Liszauer et al. [20]	M	28 y	RLL	Ulcerated lesion	4 m	Local excision	Died at 11 m
Scott et al. [21]	F	50 y	LUL	Papillomatous nodules	>7 m	Local excision/amputation	Died at 37 m
Taswell et al. [22]	M	17 y	LUL	Ulcerated lesion	Not stated	Disarticulation	Died at 24 m
Bunch [23]	F	13 y	UL	Not stated	Not stated	Interscapular amputation	Died at 1 y
Finlay-Jones [24]	M	34 y	LLL	Ulcerated blue tumor	Not stated	Excision and combined therapy	Died at 31 m
Merrick et al. [25]	M	52 y	LUL	Swelling/blue nodules	6 m	Wide local excision/amputation/radiotherapy	Died at 36 m
Mackenzie [26]	M	64 y	RLL	Nodules/ulceration	2 m	Hindquarter amputation/radiotherapy	Alive at 24 m
Dubin et al. [27]	F	29 y	LUL	Ulcerated lesion	3 y	Disarticulation	Died at 45 m
Laskas et al. [28]	F	85 y	RUL	Purple papules and blisters/ulceration	10 m	Palliative midhumeral amputation	Died at 14 m
Banathy et al. [29]	F	50 y	LUL	Purple nodules	Not stated	Midhumeral amputation	Died at 26 m
Sordillo et al. [30]	F	23 y		Not stated	Not stated	Hemipelvectomy	Alive at 19 y
Bostrom et al. [31]	F	19 y	RUL	Infected lesion	4 m	Amputation/disarticulation	Died at 12 m
	F	10 y	LLL	Blue/red nodules	8 m	Amputation/disarticulation	Alive at 10 m
Offori et al., 1993 [12]	F	43 y	LLL	Blue/purple nodules	2 m	Amputation	Alive at 9 m
Andersson et al. [32]	M	35 y	RL	Blue nodules	Not stated	Hemipelvectomy, chemotherapy	Not stated
Cerri et al. [33]	F	42 y	Pubis	Ecchymotic plaque	Not stated	Hemipelvectomy, radiotherapy	Not stated
Bernardi et al. [18]	F	4 y	LLL	Ulcerated lesion/blue nodules	1 y	Local excision	Alive at 14 m
Deyrup et al., 2011 [14]	F	2 y	Foot	Not stated	Not stated	Local excision	Died at 1 y
Present case	F	21 y	Groin	Purple/reddish infiltrated and ulcerated mass	10 m	Local excision	Died at 3 m

LLL: left lower limb; RLL: right lower limb; LUL: left upper limb; RUL: right upper limb; m: months; and y: years.
